# Cryptosporidiosis Outbreaks — United States, 2009–2017

**DOI:** 10.15585/mmwr.mm6825a3

**Published:** 2019-06-28

**Authors:** Radhika Gharpure, Ariana Perez, Allison D. Miller, Mary E. Wikswo, Rachel Silver, Michele C. Hlavsa

**Affiliations:** ^1^Division of Foodborne, Waterborne, and Environmental Diseases, National Center for Emerging and Zoonotic Infectious Diseases, CDC; ^2^Epidemic Intelligence Service, CDC; ^3^Oak Ridge Institute for Science and Education, Oak Ridge, Tennessee; ^4^Eagle Medical Services, LLC, Atlanta, Georgia; ^5^Division of Viral Diseases, National Center for Immunization and Respiratory Diseases, CDC.

*Cryptosporidium* is a parasite that causes cryptosporidiosis, a profuse, watery diarrhea that can last up to 3 weeks in immunocompetent patients and can lead to life-threatening malnutrition and wasting in immunocompromised patients.* Fecal-oral transmission can occur by ingestion of contaminated recreational water, drinking water, or food, or through contact with infected persons or animals. For the period 2009–2017, public health officials from 40 states and Puerto Rico voluntarily reported 444 cryptosporidiosis outbreaks resulting in 7,465 cases. Exposure to treated recreational water (e.g., in pools and water playgrounds) was associated with 156 (35.1%) outbreaks resulting in 4,232 (56.7%) cases. Other predominant outbreak exposures included contact with cattle (65 outbreaks; 14.6%) and contact with infected persons in child care settings (57; 12.8%). The annual number of reported cryptosporidiosis outbreaks overall increased an average of approximately 13% per year over time. Reversing this trend will require dissemination of prevention messages to discourage swimming or attending child care while ill with diarrhea and encourage hand washing after contact with animals. Prevention and control measures can be optimized by improving understanding of *Cryptosporidium* transmission through regular analysis of systematically collected epidemiologic and molecular characterization data.

A cryptosporidiosis outbreak was defined as two or more cases epidemiologically linked to a common source by location and time of exposure.^†^ Public health officials in the 50 states, the District of Columbia, U.S. territories,^§^ and Freely Associated States^¶^ voluntarily report outbreaks to CDC via the National Outbreak Reporting System (NORS). This report summarizes data from outbreak reports submitted to NORS by February 6, 2019, for which at least one etiology was *Cryptosporidium* and earliest illness onset date occurring during 2009 (the first year of NORS reporting) through 2017 (the most recent year for which data were available). NORS outbreak reports include data on etiology; counts of primary cases, hospitalizations, and deaths; transmission mode; exposures and settings; molecular characterization; and earliest illness onset date. Negative binomial regression analyses were conducted to assess trends in annual outbreak counts using SAS (version 9.4; SAS Institute).

For the period 2009–2017, public health officials from 40 states and Puerto Rico voluntarily reported to CDC 444 cryptosporidiosis outbreaks, resulting in 7,465 cases, 287 hospitalizations, and one death (Table). During this period, the eight Great Lake states** reported 254 (57.2%) cryptosporidiosis outbreaks, resulting in 3,335 (44.7%) cases (Figure 1). Exposure to treated recreational water was associated with 156 (35.1%) outbreaks, resulting in 4,232 (56.7%) cases and 183 (63.8%) hospitalizations. The most frequently implicated recreational water venues included pools (100 outbreaks; 64.1%), kiddie/wading pools (11; 7.1%), and water playgrounds (10; 6.4%). Twenty-three (14.7%) outbreaks were associated with multiple recreational water venues (e.g., multiple pools or water playgrounds). Among outbreaks associated with treated recreational water, the median case count was nine (range = 2–638). Among the 288 (64.9%) outbreaks not associated with treated recreational water, the median case count was five (range = 2–205).

**TABLE:** Cryptosporidiosis outbreaks (N = 444), cases, and hospitalizations, by mode of transmission and exposure — 40 states and Puerto Rico, 2009–2017

Transmission mode	No. (%)		
	Outbreaks	Cases	Hospitalizations
**All modes**	444 (100)	7,465 (100)	287 (100)
**Waterborne, exposure source**	183 (41.2)	5,015 (67.2)	194 (67.6)
Recreational water			
Treated (e.g., pool)	156	4,232	183
Untreated (e.g., lake)	14	263	3
Drinking water	8	339	3
Other*	5	181	5
**Person-to-person, exposure setting**	88 (19.8)	754 (10.1)	24 (8.4)
Child day care	57	418	11
Private home/Residence	15	66	5
Long-term care/Assisted living facility	2	148	0
School/College/University	2	18	1
Other^†^	8	85	6
Undetermined^§^	4	19	1
**Animal contact, reservoir**	86 (19.4)	788 (10.6)	34 (11.8)
Cattle	65	549	19
Goats	9	99	7
Sheep	1	5	0
Multiple species	4	105	6
Undetermined^§^	7	30	2
**Foodborne, vehicle**	22 (5.0)	283 (3.8)	11 (3.8)
Milk, unpasteurized	9	52	4
Apple cider, unpasteurized	4	36	1
Fresh produce^¶^	2	14	1
Undetermined^§^	7	181	5
Environmental contamination**	2 (0.5)	9 (0.1)	1 (0.3)
**Unknown^††^**	63 (14.2)	616 (8.3)	23 (8.0)

* Waterborne outbreaks were categorized as other if the outbreak was associated with environmental exposures to water (i.e., water from a source other than a recreational venue or drinking water system) or with undetermined exposures to water (i.e., evidence to implicate a single type of water exposure was insufficient).

^†^ Person-to-person outbreaks categorized as other involved the following settings: camp (one), festival/fair (one), hospital (one), hotel/motel (one), and other unspecified (four). The person-to-person outbreak associated with a hospital setting resulted in one reported death.

^§^ Outbreaks were categorized as undetermined if mode of transmission was identified but specific water exposure, setting of person-to-person exposure, animal reservoir, food vehicle, or fomite was not identified or reported.

^¶^ One fresh produce–associated foodborne outbreak was associated with strawberries, the other with kale.

** Outbreaks were categorized as environmental cryptosporidiosis outbreaks if *Cryptosporidium* was transmitted through contact with fomites, such as dirty linens or high-touch bathroom surfaces.

^††^ Outbreaks were categorized as having an unknown transmission mode if evidence to implicate one specific primary mode of transmission was insufficient.

**FIGURE 1:**
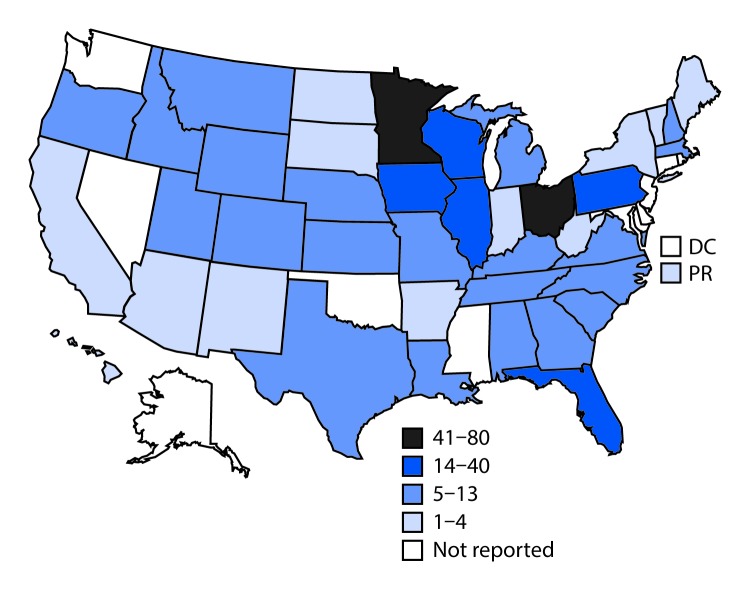
Reported cryptosporidiosis outbreaks (N = 444), by exposure jurisdiction* — United States, 2009–2017^†^ **Abbreviations:** DC = District of Columbia; PR = Puerto Rico. * Exposure jurisdictions are states, DC, and PR. ^†^ These numbers are largely dependent on public health capacity and reporting requirements, which vary across jurisdictions and do not necessarily indicate the actual occurrence of cryptosporidiosis outbreaks in a given jurisdiction.

Among all 444 outbreaks, 65 (14.6%) were associated with contact with cattle, resulting in 549 cases; 57 (12.8%) were associated with contact with infected persons in child care settings, resulting in 418 cases. Among the 22 foodborne outbreaks, nine (40.9%) were associated with unpasteurized milk and four (18.2%) with unpasteurized apple cider. The mode of transmission was unknown for 63 (14.2%) outbreaks; the predominant settings included private homes/residences (18; 28.6%) and child care (12; 19.0%). Molecular characterization data were available for 67 (15.1%) outbreaks, only one (1.5%) of which had unknown mode of transmission.

Negative binomial regression analysis indicated that during 2009–2017, the overall annual number of reported cryptosporidiosis outbreaks increased an average of 12.8% per year (95% confidence interval [CI] = 7.6%–18.0%) (Figure 2). The annual number of reported treated recreational water–associated outbreaks increased an average of 14.3% (95% CI = 3.4%–25.2%) per year during 2009–2016 (p = 0.010); however, because of a decline in reported outbreaks in 2017, no trend was found for the annual number of treated recreational water–associated outbreaks during 2009–2017 (p = 0.293). During 2009–2017, the annual number of reported outbreaks associated with contact with cattle increased an average of 20.3% (95% CI = 9.2%–31.4%) per year, and the annual number of reported outbreaks associated with contact with infected persons in child care settings increased an average of 19.7% (95% CI = 8.8%–30.5%) per year. During 2009–2017, the overall number of reported cryptosporidiosis outbreaks by month peaked during July–August, the number associated with treated recreational water peaked in June–August, the number associated with cattle contact peaked during March–May, and those associated with contact with infected persons in child care settings peaked during July–September (Figure 2).

**FIGURE 2:**
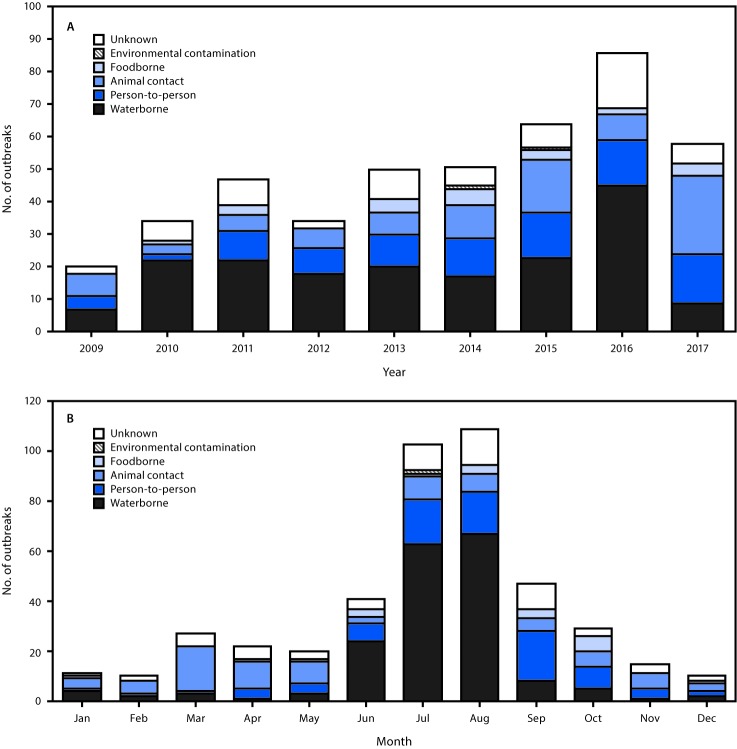
Reported cryptosporidiosis outbreaks (N = 444), by mode of transmission* and year of earliest illness onset date (A) and month of earliest illness onset date (B) — United States, 2009–2017 * Transmission modes were categorized as follows: *Unknown* if insufficient evidence to implicate one specific primary mode of transmission; *Environmental contamination* if transmitted through exposure to a contaminated environment not attributable to foodborne, waterborne, person-to-person, or animal contact transmission; *Foodborne* if transmitted by consumption of contaminated food or non-water beverages; *Animal contact* if transmitted through contact with animals or their living environments; *Person-to-person* if transmission occurred from direct contact with an infected person, their bodily fluids, or by contact with the local environment where the exposed person was simultaneously present; and *Waterborne* if transmission occurred via ingestion, inhalation, contact, or another exposure to water (e.g., treated or untreated recreational water, drinking water [including bottled water], or an environmental or indeterminate water source). https://www.cdc.gov/nors/forms.html.

## Discussion

The 444 outbreaks characterized in this report highlight the public health importance of *Cryptosporidium,* which is the leading etiology of waterborne outbreaks (*1*) and the third leading etiology of enteric infections attributable to animal contact (*2*) in the United States. In part, this is because *Cryptosporidium* oocysts are immediately infectious upon excretion, are excreted in numbers multiple orders of magnitude higher than the human infectious dose (≤10 oocysts), and are extremely tolerant to chlorine. These factors should be considered in the development of effective cryptosporidiosis prevention measures.

The number of treated recreational water–associated outbreaks caused by *Cryptosporidium* drives the summer seasonal peak in both waterborne cryptosporidiosis outbreaks and cryptosporidiosis outbreaks overall. A treated recreational water–associated cryptosporidiosis outbreak can result in hundreds or thousands of cases, because 1) an infected swimmer can excrete 10^7^–10^8^ oocysts in one diarrheal incident in the water (*3*); 2) *Cryptosporidium* oocysts can survive >7 days at CDC-recommended concentrations of >1 ppm free available chlorine (*4*); and 3) swimmers might use multiple recreational water venues.

The summer seasonal peak of cryptosporidiosis outbreaks associated with child care is similar to that of treated recreational water–associated outbreaks. Contributing factors include 1) cryptosporidiosis disproportionately affects children aged 1–4 years (*5*); 2) young children, who have no or limited toileting skills and who ingest recreational water, often use one or more kiddie/wading pools, water playgrounds, and other treated recreational water venues; and 3) chlorine (or bleach) is the primary barrier to pathogen transmission in child care facilities. Consequently, community-wide cryptosporidiosis outbreaks, in which an outbreak associated with a single treated recreational water venue evolves into one associated with multiple venues and settings (e.g., child care facilities), have been documented (*6*). Thus, primary prevention of *Cryptosporidium* contamination is important. CDC recommends not swimming or attending child care if ill with diarrhea and not swimming for an additional 2 weeks after diarrhea has resolved.^††^ If a cryptosporidiosis outbreak occurs, substantial decontamination measures are needed, including hyperchlorinating^§§^ public treated recreational water venues (e.g., at a hotel, apartment complex, or waterpark) and using hydrogen peroxide^¶¶^ to disinfect surfaces in child care settings to inactivate *Cryptosporidium* oocysts.

*Cryptosporidium* contamination can be unavoidable and widespread in environments where ruminants such as cattle, goats, and sheep live. *Cryptosporidium* transmission from preweaned calves to humans has been well documented, and the spring seasonal peak in outbreaks associated with contact with cattle coincides with the spring calving season (*7*). Bovine calves can shed >10^10^ oocysts daily (*8*). To minimize further contamination and risk for infection, CDC recommends hand washing*** after coming in direct or indirect contact with ruminants or their living environments. Additional preventive measures include, but are not limited to, removing clothing and shoes worn in the animals’ living environment before entering other environments (e.g., a home) to reduce risk for cross-contamination.

*Cryptosporidium* caused 13 outbreaks associated with unpasteurized milk or apple cider during 2009–2017. Outbreak sources might include contaminated udders, apples, or processing equipment. CDC recommends consumption of pasteurized milk and apple cider because of the risk for infection from unpasteurized products in general and the risk for severe illness in young children, pregnant women, and immunocompromised persons.^†††^

The findings in this report are subject to at least five limitations. First, the outbreaks described in this report likely underestimate the actual number of cryptosporidiosis outbreaks, and the reported number of cases likely underestimate the actual magnitude of individual outbreaks. Second, the advent of multipathogen molecular testing panels, which include *Cryptosporidium*, could have contributed to the increase in reported outbreaks in recent years. Third, requirements and capacity to detect, investigate, and report outbreaks vary across jurisdictions. Thus, it is unclear if approximately half of the outbreaks actually occurred in the Great Lakes states; further investigation is warranted. Fourth, only two outbreaks were determined to be the result of transmission by environmental contamination; this might be because of difficulties inherent to implicating fomites as an outbreak source. Finally, only 67 NORS outbreak reports included molecular characterization data, precluding analysis of mode of transmission by *Cryptosporidium* species and genotypes.

Reversing the increasing trends in annual numbers of reported cryptosporidiosis outbreaks overall and those associated with treated recreational water, contact with cattle, or contact with infected persons in child care settings will require implementing effective prevention measures.^§§§^ Approximately 40 *Cryptosporidium* species have been identified to date, of which 17 species and four additional genotypes have been reported to infect humans (*9*). Most *Cryptosporidium* species and genotypes cannot be distinguished by traditional diagnostic tests (microscopy or immunoassays). Therefore, advancing molecular characterization methods, such as those used by CryptoNet, the first U.S. molecularly based surveillance system for a parasitic disease, might help optimize efforts to prevent cryptosporidiosis. Given that individual species, genotypes, and subtypes can have unique host ranges, molecular characterization can provide insight into outbreak exposures and sources. CryptoNet has already demonstrated its ability to elucidate *Cryptosporidium* transmission chains when used in investigations of treated recreational water–associated outbreaks (*10*) and has the potential to do the same for investigations of cryptosporidiosis outbreaks not associated with treated recreational water.

**Summary**

**What is already known about this topic?**

*Cryptosporidium* is the leading cause of outbreaks of diarrhea linked to water and the third leading cause of diarrhea associated with animal contact in the United States.

**What is added by this report?**

During 2009–2017, 444 cryptosporidiosis outbreaks, resulting in 7,465 cases were reported by 40 states and Puerto Rico. The number of reported outbreaks has increased an average of approximately 13% per year. Leading causes include swallowing contaminated water in pools or water playgrounds, contact with infected cattle, and contact with infected persons in child care settings.

**What are the implications for public health practice?**

To prevent cryptosporidiosis outbreaks, CDC recommends not swimming or attending child care if ill with diarrhea and recommends hand washing after contact with animals.

## Footnotes

* https://www.cdc.gov/parasites/crypto/index.html.

† https://www.cdc.gov/nors/forms.html.

§ American Samoa, Guam, Northern Mariana Islands, Puerto Rico, and the U.S. Virgin Islands.

¶ Federated States of Micronesia, Marshall Islands, and Palau.

** Illinois, Indiana, Michigan, Minnesota, New York, Ohio, Pennsylvania, and Wisconsin.

†† https://www.cdc.gov/parasites/crypto/childcare/prevent.html;https://www.cdc.gov/healthywater/swimming/swimmers/steps-healthy-swimming.html.

§§ At water pH ≤7.5 and temperature ≥77°F (25°C), 2- to 3-log10 (99%–99.9%) *Cryptosporidium* inactivation can be achieved by raising the free available chlorine concentration for a prolonged period (https://www.cdc.gov/healthywater/swimming/pdf/fecal-incident-response-guidelines.pdf). CDC does not recommend hyperchlorinating residential venues (e.g., those in a backyard).

¶¶ Do not mix hydrogen peroxide and bleach solutions; the two can react violently. If the health department instructs a child care facility to disinfect surfaces with both a bleach solution and hydrogen peroxide, facility personnel should first clean the surface and then disinfect it with the bleach solution, thoroughly rinse it with water, soak it with hydrogen peroxide for 20 minutes, and thoroughly rinse it with water. https://www.cdc.gov/parasites/crypto/childcare/outbreak.html.

*** Alcohol-based hand sanitizers do not effectively inactivate *Cryptosporidium*. Furthermore, hand sanitizers might not effectively inactivate pathogens, in general, when hands are visibly dirty or greasy. https://www.cdc.gov/handwashing/index.html.

††† https://www.cdc.gov/foodsafety/rawmilk/raw-milk-index.html.

§§§ https://www.cdc.gov/parasites/crypto/gen_info/prevention-general-public.html.
